# Homœopathic Statistics

**Published:** 1850-10

**Authors:** 


*Homœopathic Statistics*—Very erroneous impressions obtain, to some extent, respecting the diffusion of the homœopathic delusion in other countries. It is thought by some that this mode of practice has secured a permanent foothold in Europe. This is asserted as an inducement for the credulous to become converts to the doctrine, the tendency to follow in the wake of European currents of opinion on some subjects being an obvious weakness of a portion of our countrymen. The following facts, communicated for the London Medical Times, exhibit the numerical ratio of homœopathic practitioners to the population, and the regular profession in Great Britain:— 1*st.* *Of London.*

The population of London amounts to about.................2,200,000

The number of medical practitioners, practising in London, whose names appear in the "London Medical Directory," is....... 2,571

The number of homœopathic practitioners, practising in London, according to the accredited "list," in the *British Journal of Homœopathy* for January, 1850, is...................... 48

Of these 48 homœopathic practitioners, 22 are *not* in the *London Medical Directory* at all; and of the 26 which remain, 10 are graduates in medicine, and 16 are surgeons or surgeon-apothecaries.

Of the ten graduates, 6 appear to have the Edinburgh degree, 1 Aberdeen and Paris, 1 Aberdeen and Turbingen, 1 Aberdeen, 1 Erlangen.

2*d.* *Of the Provinces.*

According to the *Provincial Medical Directory,* there are of medical practitioners, practising in the provinces........... 8,327

According to the "homœopathic list," already referred to, there are, of homœopathic practitioners, practising in the provinces, 52

Of these 52 homœopathic practitioners, 16 are *not* in the *Provincial Medical Directory* at all; 4 are in it, but their qualifications are not vouched for by the Editor of the Directory; and of the remaining 32 whose names appear in the Directory, 18 are graduates in medicine, and 14 are surgeons or surgeon-apothecaries.

Of the 18 graduates, 13 possess the Edinburgh degree, 3 St. Andrews, 1 Glasgow, 1 is an Est. Lic. Lond. Coll. Ph.

There appear, therefore, to be in England, about 10,898 medical practitioners; but suppose that we make a liberal deduction from this number of 898, as practitioners of doubtful license, and make the number of legalized practitioners 10,000, instead of 10,898; then out of this number appears the insignificant proportion of 28 graduates in medicine, and 45 general practitioners, who call themselves homœopaths, and who profess to practice as such.

By the foregoing statistics it is apparent that sugar pellets are not in very general repute in Great Britain. They are just now much more in vogue in this country. But the truth, probably, is, that having traveled to England before visiting the United States, the system has had its day, and is now in the sear and yellow leaf of whatever popularity it may have had heretofore in that kingdom. That it will have its decline and fall in this country in a few years, it requires but little shrewdness to foresee. But it may be doubted if this result will denote any abatement of the spirit of quackery. The same credulity and love of the marvelous which have fostered homœopathy, and other impositions, will remain. A portion of every community will still insist on being duped; and, doubtless, the fertility of invention will be adequate to supply a worthy successor to the fictions of Hahnemann.

We have no disposition to scold or fret about homœopathy. Like other dispensations of Providence, it has its specific objects to accomplish, and some of these may possibly, in a measure, compensate for the sacrifice of life and health incident to its career. It furnishes some illustrations of the progress and results of diseases uninfluenced by medication. These would be truly valuable if confidence could be placed in the competency and integrity of homœopathic practitioners. It may perhaps exert some effect in preparing the minds of people not to consider medical advice and the administration of active remedies as indissolubly connected as cause and effect. To remove this popular error, has for many years past been the aim and tendency of medical practice. The latter is sometimes attributed mainly to homœopathy. It would be nearer the truth to reverse the proposition. Another reflection which should reconcile us somewhat to the absurdities of homœopathy is, that if this delusion did not exist, the credulity of the public might be exposed to some other form of deception more disastrous in its consequences. Finally, there is another consideration which applies to this, as to all the phases of medical quackery. This is, that although the world may not grow wiser by experience, individuals do. On the principle embodied in the homely apothegm, "a child once burned, ever after dreads the fire," they who once discover the folly or danger of a popular medical delusion, are not apt to be duped a second time. These persons thereafter will be found to be the firmest friends of legitimate medicine. This consideration may prove a succedaneum for practice temporarily lost, and should also dictate the line of policy to pursue with those who choose to make trial of a fashionable humbug.

We trust that we have not laid ourselves open to the charge of being an apologist for homœopathy by these remarks. Regarding it as one of the entities of the day for which we are in no wise responsible, we have indulged a few of the thoughts suggesting themselves at the moment of writing, which pertain to the cultivation of a philosophical endurance of those of the evils and absurdities of human life which it were bootless to attempt to remedy.

